# Erianin induces G2/M-phase arrest, apoptosis, and autophagy via the ROS/JNK signaling pathway in human osteosarcoma cells *in vitro* and *in vivo*

**DOI:** 10.1038/cddis.2016.138

**Published:** 2016-06-02

**Authors:** H Wang, T Zhang, W Sun, Z Wang, D Zuo, Z Zhou, S Li, J Xu, F Yin, Y Hua, Z Cai

**Affiliations:** 1 Department of Orthopaedics, Shanghai First People's Hospital, Shanghai Jiao Tong University, Shanghai, China; 2 Department of Orthopaedics, Yangpu Hospital, Tongji University, Shanghai, China; 3 Department of Orthopaedics, Shanghai Tenth Peoples's Hospital, Tongji University, Shanghai, China; 4 Department of Orthopaedics, Shanghai East Hospital, Tongji University, Shanghai, China

## Abstract

Erianin, a natural product derived from *Dendrobium chrysotoxum*, has exhibited potential antitumor activity in various malignancies, including hepatocarcinoma, melanoma, and promyelocytic leukemia. Here we explored the effects of erianin on osteosarcoma (OS) *in vitro* and *in vivo* and further elucidated the underlying molecule mechanisms. In this study, we found that erianin potently suppressed cell viability in various OS cell lines. Treatment with erianin induced G2/M-phase arrest, apoptosis, and autophagy in OS cells. Further studies showed that erianin-induced apoptosis and autophagy was attributed to reactive oxygen species (ROS), as *N*-acetyl cysteine (NAC), an ROS scavenger, attenuated them. Moreover, we found that erianin induced activation of c-Jun N-terminal kinase (JNK) signal pathway, which was also blocked by NAC. Downregulation of JNK by its specific inhibitor SP600125 could attenuate apoptosis and autophagy induced by erianin. Finally, erianin *in vivo* markedly reduced the growth with little organ-related toxicity. In conclusion, erianin induced cell cycle G2/M-phase arrest, apoptosis, and autophagy via the ROS/JNK signaling pathway in human OS. In light of these results, erianin may be a promising agent for anticancer therapy against OS.

Osteosarcoma (OS), the most common primary bone tumor, is derived from primitive bone-forming mesenchymal cells, which produces osteoid and/or immature bone.^1^ Approximately 2–3 patients per million were diagnosed with OS annually. The prevalence is predominant in adolescence, of which the annual incidence is 8–11 per million at 15–19 years of age.^2^ Because of the multi-agent, dose-intensive chemotherapy in conjunction with improved surgical techniques, the 5-year survival rate of patients with OS has been improved to 60–70%.^3^ Unfortunately, this cure rate has not increased over the past 25–30 years. Therefore, continuing research into new treatment approaches and drugs is urgently needed.

The combretastatins are a group of antimitotic agents isolated from the bark of the South African tree *Combretum caffrum*. Structurally, combretastatins consist of two substituted aromatic (aryl) rings (rings A and B) linked by a two-carbon alkene bridge.^4^ The combretastatins are naturally very potent anticancer agents with activity in the low nanomolar range.^5^ Combretastatin A-4 (CA-4) is one of the most potent antitumor agents, which shows strong cytotoxicity against a variety of cancer cells, including multi-drug-resistant cancer cell lines.^6^ CA-4 has a high affinity for tubulin and destabilizes the tubulin polymers of the cytoskeleton, resulting in morphological changes.^7, 8^ In addition, the CA-4 derivatives also evolved as a prevalent strategy to complement established chemotherapeutics for the treatment of solid tumors.

Structurally similar to CA-4, erianin can induce microtubule disassembly resulting in a dramatic cytotoxic effect on various human cancer cells. Erianin is a natural product derived from *Dendrobium chrysotoxum* and has been used as an analgesic in traditional Chinese medicine. Previous studies have demonstrated the antitumor activity of erianin against a variety of human cancer cells, including human hepatocarcinoma Bel7402 cells,^9^ human melanoma A375 cells,^9^ and human promyelocytic leukemia HL-60 cells.^10^ However, whether erianin suppresses the growth of human OS and its related molecular mechanism have not yet been investigated.

Many cytotoxic agents and/or microtubule-targeting agents inhibit tumor cell proliferation by causing cell cycle G0-, S-, or G2/M-phase arrest.^11, 12, 13^ The G2 checkpoint prevents cells from entering mitosis when DNA is damaged and ensures the propagation of error-free copies of the genome to each daughter cell. Cdk1/Cyclin B1 complex controls the cell cycle progression from G2 phase to the M phase by regulating the phosphorylation or dephosphorylation of proteins.^14^ In addition, actin remodeling in coordination can ensure proper execution of G2/M checkpoint arrest and is crucial for entry into mitosis.^15, 16^

Cell death is a hallmark of cancer that can be classified according to morphological differences. Apoptosis, the best defined form of programmed cell death (PCD), is characterized by specific morphological and biochemical changes of dying cells, including cell shrinkage, nuclear condensation and fragmentation, dynamic membrane blebbing, and loss of adhesion to neighbors or to extracellular matrix.^17, 18^ Autophagy, or type II PCD, is a lysosomal degradation procedure by which excessive or dysfunctional eukaryotic cellular components are transported into lysosomes to be digested.^18, 19^ The functional relationship between apoptosis and autophagy is complex, and the two phenomena jointly seal the fate of the cell.^20^ Therefore, further investigations are required for the apoptosis–autophagy crosstalk, which may provide novel concepts and new targeted agents for cancer therapy.

Reactive oxygen species (ROS) has been described as a heterogeneous group of diatomic oxygen from free and non-free radical species and has important roles in biochemical functions, including apoptosis and autophagy. ROS triggers apoptosis by causing various cellular stresses, including DNA damage and microtubule disruption mediated by various signal transducers.^21, 22^ Several apoptotic effectors are redox-sensitive, such as caspases, Bcl-2, and cytochrome *c*, and their functions are markedly regulated by cellular ROS.^23^ Recent studies have showed that antioxidative agents abolished the formation of autophagosomes and the consequent degradation of proteins.^24^ Starvation-induced autophagy, in turn, resulted in ROS production and DNA damage by targeting the poly (ADP-ribose) polymerase (PARP) signaling pathway.^25^

ROS have been demonstrated as an inducer or mediator for the activation of MAPK family members, including c-Jun N-terminal kinase (JNK), p38, and ERK1/2.^26^ JNK, also known as stress-activated protein kinase of MAPK family, is a key regulator of a variety of cellular events, including apoptosis and autophagy. The function of JNK in apoptosis is complex, depending on cell type, nature of the death stimulus, duration of its activation and the activity of other signaling pathways.^27, 28, 29^ In addition to apoptosis, JNK also contributes to autophagic induction in response to stress signals, for instance, incidences of nutrition deficiency,^30^ cytokine and growth factor decreases,^31^ and chemotherapy drugs.^32^

In the current study, we elucidated that erianin induced G2/M-phase arrest, apoptosis, and autophagy in human OS cells, which was mediated by ROS induction, leading to activation of JNK/c-Jun signaling cascades. Furthermore, we investigated that erianin administration inhibited tumor growth using *in vivo* tumor xenograft model. Collectively, our data suggest that erianin is a promising antitumor agent by modulating the ROS/JNK signaling pathway for OS.

## Results

### Erianin inhibits cell proliferation and induces cell cycle G2/M arrest in OS cells

**Figure 1:**
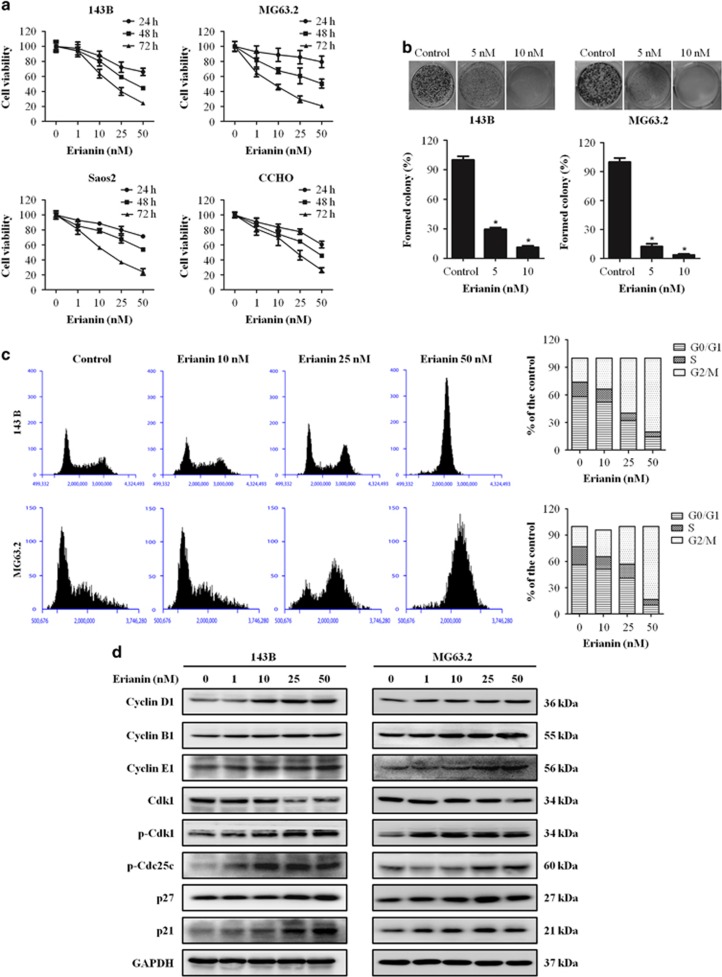
Erianin inhibits cells proliferation and induces G2/M arrest in human OS cells. (**a**) CCK8 assay was used to assessed OS cell proliferation. OS cell viability following treatment with the various concentrations of erianin for 24, 48, and 72 h. (**b**) Colony-formation assay was performed in 143B and MG63.2 cells with and without erianin treatment. (**c**) Erianin induces G2/M cell cycle arrest. 143B and MG63.2 cells were treated with control and erianin (10, 25, and 50 nM) for 24 h. The distribution of cell cycle was assessed by flow cytometry. The percentage of cells in each phase is showed as mean±S.D. from three independent experiments. (**d**) 143B and MG63.2 cells were exposed to erianin for 24 h. The expression of cell cycle-regulated proteins were analyzed by western blotting. *P<0.05, significantly different compared with control

To investigate the inhibitory effects and cytotoxicity of erianin in OS cells, 143B, MG63.2, Saos2, and CCHO were treated by various concentrations of erianin for 24, 48, and 72 h, followed by Cell Counting Kit-8 (CCK8) assay. We found that erianin decreased OS cell viability in a time and dose-dependent manner (Figure 1a). The IC50 values were 58.19 nM (24 h), 40.97 nM (48 h), and 26.77 nM (72 h) for 143B cells, while the IC50 values for MG63.2 were 88.69 nM (24 h), 44.26 nM (48 h), and 17.20 nM (72 h). In addition, the antiproliferation effect of erianin in OS cells 143B and MG63.2 was confirmed by colony-formation assay. Results demonstrated that erianin treatment significantly reduced the number of colonies in a dose-dependent manner when compared with untreated cells (Figure 1b). These results demonstrated that erianin treatment inhibited the proliferation of OS cells.

To verify the causal relation of cell proliferation inhibition and cell cycle arrest, the cell cycle distribution was analyzed. Erianin increased cell number at G2/M phase after 24 h treated with raising concentration, accompanied by decreased cell number at S and G0/G1 phases in 143B and MG63.2 cells (Figure 1c). Furthermore, cell cycle-regulating pathways were measured by western blotting, the expression of Cyclin B1, phospho-Cdk1, phospho-Cdc25c, p21, and p27 were upregulated, and the level of Cdk1 was downregulated (Figure 1d). All these data indicated that erianin triggered G2/M-phase arrest by regulating cell cycle-related proteins.

### Erianin induces apoptosis of OS cells

**Figure 2:**
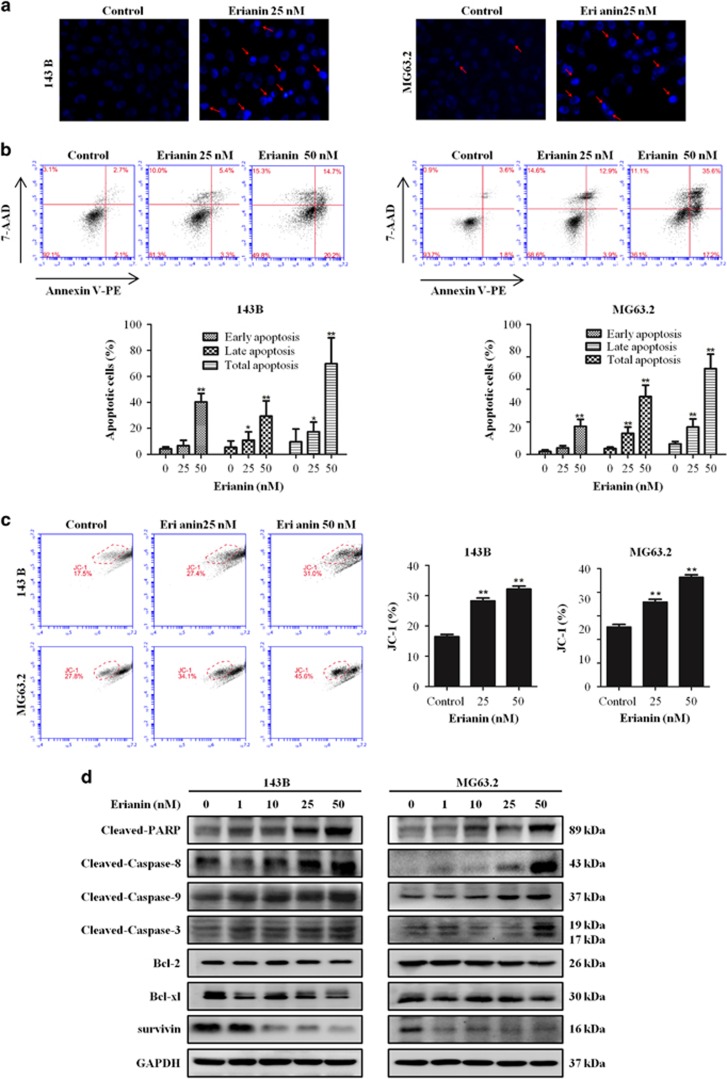
Erianin induces apoptosis in OS cells. (**a**) Apoptotic nuclear morphology changes induced by erianin were assessed by Hoechst 33342 staining and visualized by fluorescence microscopy. Arrows indicate chromatin condensation and nuclear fragmentation. (**b**) Cells were treated with increased concentrations of erianin for 24 h. Cells were processed by flow cytometry using Annexin V-PE/7-AAD (7-aminoactinomycin D) staining and analyzed by flow cytometry. The histograms indicate that the percentage of early apoptosis, late apoptosis, and total apoptosis. The percentage of apoptosis cells is shown as mean±S.D. from three independent experiments. (**c**) The mitochondrial membrane potential after erianin treatment were measured using JC-1 staining by flow cytometry. The histograms indicate the ratio of green in JC-1 fluorescence. Results are shown as mean±S.D. from three independent experiments. (**d**) 143B and MG63.2 cells were incubated with erianin for 24 h. Cell lysates were prepared and analyzed by western blotting for cleaved PARP, caspase-8, -9, and -3, Bcl-2, Bcl-xl, and survivin. *P<0.05 and **P<0.001, significantly different compared with control

We further explored the effects of erianin on apoptosis and cell death in OS cells. Hoechst 33342 staining was used to estimate erianin-dependent changes in cell morphology. Results demonstrated the occurrence of cell shrinkage, chromatin condensation, and nuclei fragmentation after treatment with erianin for 24 h (Figure 2a). Flow cytometric analysis via Annexin V-PE/7-AAD was performed. We found that a significant increase of the number of apoptotic cells was observed in cells after exposed to erianin in a dose-dependent manner (Figure 2b). Next we used the fluorescent mitochondrial probe JC-1 to measure mitochondrial membrane potential to confirm the involvement of mitochondrial in the induction of apoptosis by erianin. After erianin treatment, an obvious shift from red to green was observed (Figure 2c), indicating that mitochondrial depolarization was induced by erianin in OS cells. To further determine whether extrinsic or intrinsic pathway mediated erianin-induced apoptosis, we investigated the expression of downstream apoptotic proteins by western blotting. Caspase-8 and -9 are initiator caspases in the extrinsic and intrinsic (mitochondrial) apoptosis pathways, respectively. As shown in Figure 2d, an obvious increase in the activation of cleavage caspase-3, -8, and -9 and of PARP and the expression of Bcl-2, Bcl-xl, and survivin were decreased (Figure 2d). Taken together, these data indicated that erianin provoked cell apoptosis by activating both the extrinsic and intrinsic pathways.

### Erianin induces autophagy of OS cells

**Figure 3:**
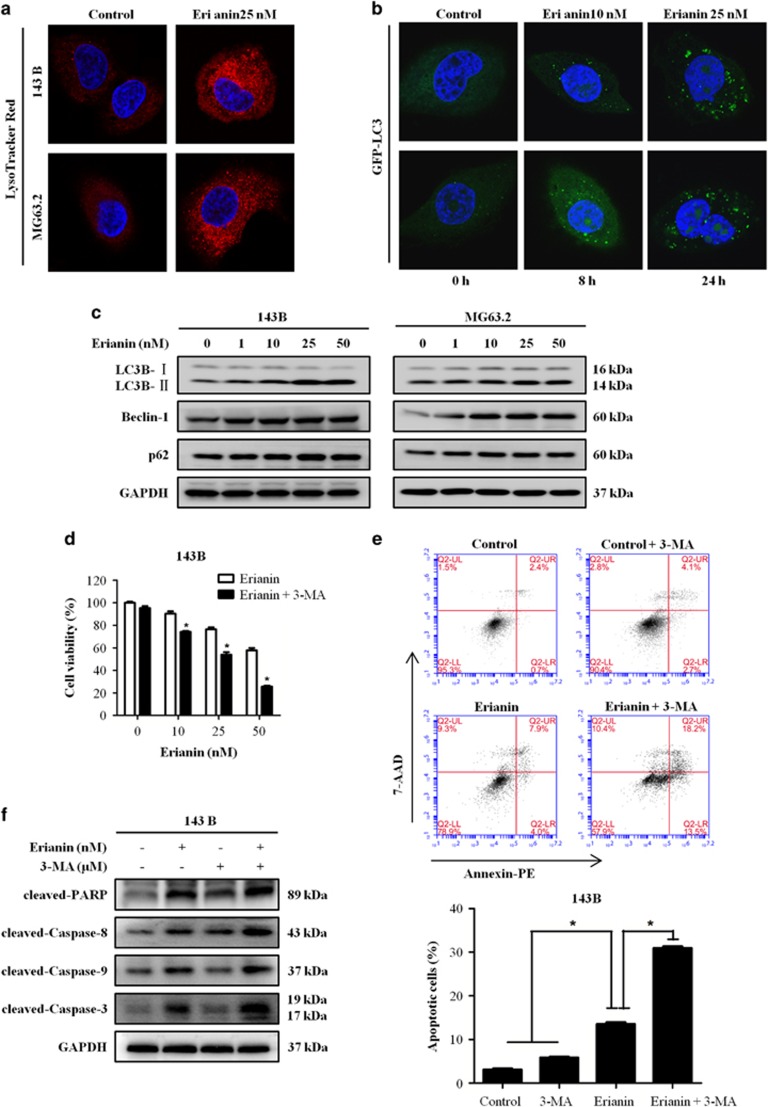
Erianin induces autophagy and inhibition of autophagy increases erianin-induced apoptosis. (**a**) Representative images of LysoTracker Red staining of OS cells following treatment with 25 nM erianin for 24 h. Red color intensity shows acidic vesicular organelles, indicating autophagosomes. (**b**) Representative micrographs of cells that shows GFP-LC3 localization. MG63.2 cells stably expressing GFP-LC3B were treated with control and the indicated concentrations of erianin for 24 h (upper panel) or 25 nM erianin for the indicated time (bottom panel). Erianin-treated cells displayed a punctate pattern of GFP-LC3 expression, representing formation of autophagosomes. (**c**) Cells were treated with various concentrations of erianin for 24 h. The expression of LC3B, Beclin-1, and p62 were analyzed by western blotting. (**d**) 143B cells were preincubated with 3-MA (5 mM) for 2 h and then treated with erianin for 24 h, followed by CCK8 assay of cell proliferation. (**e**) 143B was preincubated with 3-MA (5 mM) for 2 h and then treated with erianin for 24 h, followed by Annexin V-PE/7-AAD (7-aminoactinomycin D) staining. The percentage of apoptosis cells is shown as mean±S.D. from three independent experiments. *P<0.05, significantly different compared with the control and 3-MA-treated group. (**f**) 143B was preincubated with 3-MA (5 mM) for 2 h and then treated with erianin for 24 h, followed by western blotting analysis of apoptosis-related proteins

As autophagy contributes to cell death, we then investigated whether erianin induces autophagy in OS cells. Autophagy is characterized by the increased acidic vesicular organelles, which are correlated with increased autophagosomes.^33^ We used the membrane acidotropic dye probe LysoTracker Red label cellular acidic compartments, including lysosomes and autolysosomes. Upon exposure to erianin for 24 h, the fluorescence intensity in 143B and MG63.2 cells exhibited an obvious increase (Figure 3a). Furthermore, we found that treating cells with erianin resulted in a significant increase in GFP-LC3 puncta formation in MG63.2 cells in both dose- and time-dependent manner (Figure 3b). To verify the above finding, we further tested the expression of several marker protein of autophagy by western blotting. Erianin treatment increased the amount of LC3B II protein and upregulated the expression of p62 and Beclin-1 (Figure 3c). Overwhelmingly, evidences show that autophagy has a dual role for therapeutic purpose in cancer, with response to protect cell survival or contribute to cell death. We used the autophagy inhibitor 3-methyladenine (3-MA) to block erianin-induced autophagy in OS cells. Consistently, the administration of 3-MA increased OS cells' sensitivity to erianin by reducing its autophagic effects and enhancing its apoptotic effects (Figures 3d–f). These data suggested that cellular reactive autophagy after erianin treatment may be protective in OS cells.

### Erianin activates JNK/c-Jun signaling pathway by inducing ROS production

**Figure 4:**
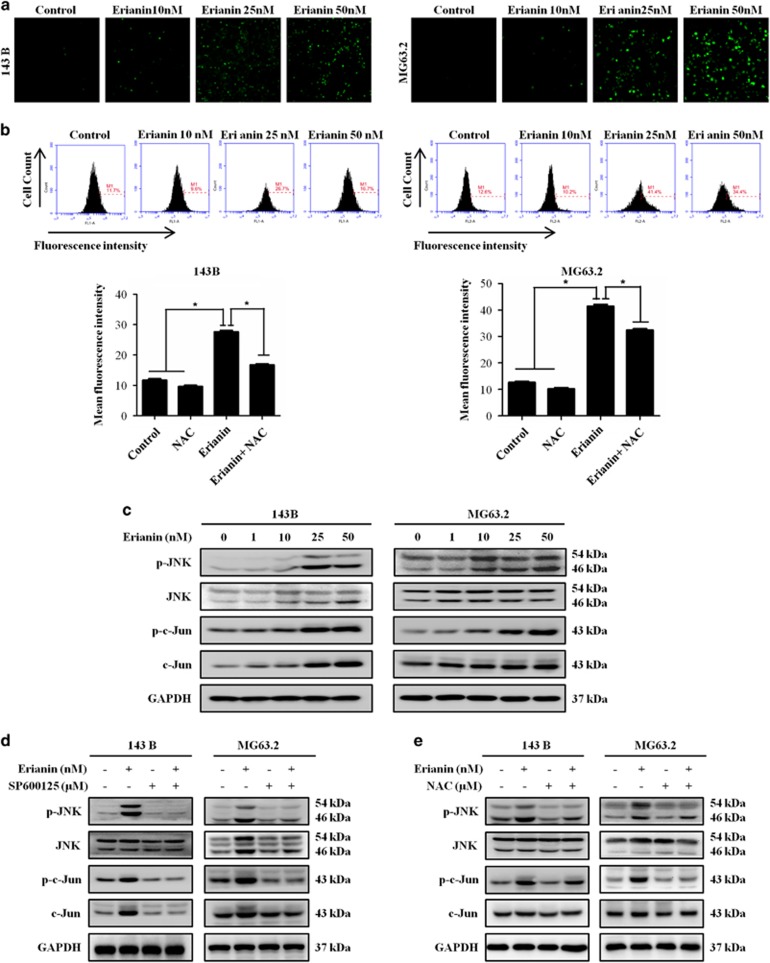
Erianin increases ROS generation and activates JNK signal pathway. (**a**) Cells were treated with increased concentrations of erianin 12 h, followed by loading with 10 *μ*M of DCFH–DA for 30 min. The level of ROS was determined by fluorescence microscopy. (**b**) Cells were preincubated with NAC (5 mM) for 2 h and then treated with erianin for 12 h. Fluorescent intensity was detected using flow cytometry. Results are presented as mean±S.D. from three independent experiments. *P<0.05, significantly different compared with the control and NAC-treated group. (**c**) Cells were treated with various concentrations of erianin for 24 h. The expression of p-JNK, JNK, p-c-Jun, and Jun were analyzed by western blotting. (**d** and **e**) 143B was preincubated with SP600125 (30 *μ*M) or NAC (5 mM) for 2 h and then treated with erianin for 24 h. Levels of p-JNK, JNK, p-c-Jun, and Jun were analyzed by western blotting

ROS functions as signaling molecules, which has important roles in biochemical functions, including apoptosis and autophagy. Therefore, the production of ROS was analyzed in erianin-treated cells by DCFH–DA staining by fluorescence microscope and flow cytometry. Figure 4a demonstrated that exposure of cells to erianin resulted in a dramatic increase in the fluorescent signal as compared with the control. We used the antioxidant *N*-acetyl cysteine (NAC) to further confirm the elevation of ROS. Addition of NAC effectively blocked erianin-induced ROS in OS cells (Figure 4b). Next we investigated the effect of erianin on JNK/c-Jun pathway. As shown in Figure 4c, erianin induced phosphorylation of JNK and c-Jun in a concentration-dependent manner. However, the presence of JNK inhibitor, SP600125, potently inhibited the activation of JNK pathway (Figure 4d). ROS has been demonstrated as an inducer or mediator for the activation of JNK/c-Jun signaling pathway. We further found that pretreatment with NAC significantly reversed the phosphorylation of JNK and c-Jun in OS cells (Figure 4e). These results revealed that erianin activated the ROS/JNK signaling pathway.

### Erianin induces apoptosis and autophagy via the activation of ROS/JNK pathway

**Figure 5:**
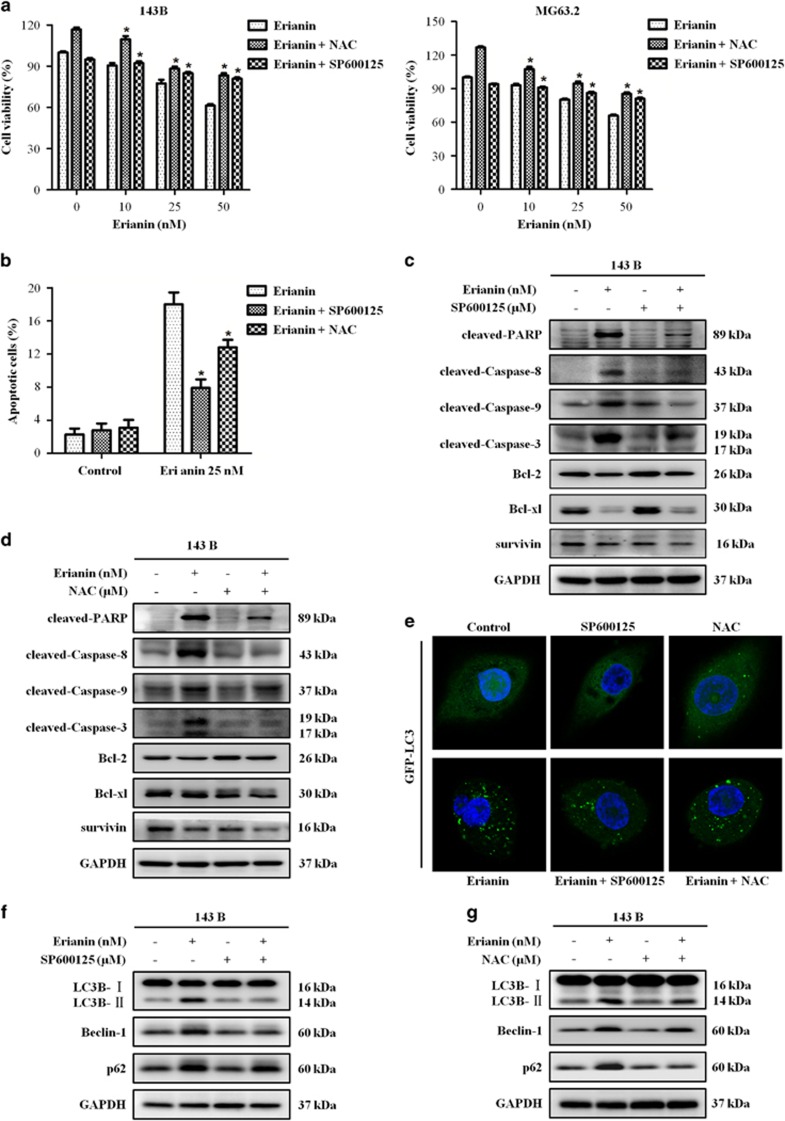
ROS/JNK pathway mediate erianin-induced apoptosis and autophagy in OS cells. Cells were preincubated with SP600125 (30 *μ*M) or NAC (5 mM) for 2 h and then treated with erianin for 24 h. (**a**) Cell viability was measured by CCK8 assay. (**b**) Cell apoptosis was evaluated by flow cytometry. Results are presented as mean±S.D. from three independent experiments. *P<0.05, significantly different compared with the control, SP600125-treated, and NAC-treated groups. (**c** and **d**) The expression of apoptosis-related proteins were measured by western blotting. (**e**) Representative micrographs of cells that shows GFP-LC3 localization. (**f** and **g**) The level of autophagy-related proteins were analyzed by western blotting

We examined whether erianin-induced apoptosis and autophagy involve ROS generation and JNK activation in OS cells. Cells were first pretreated with NAC and SP600125, respectively, before they were treated with erianin for additional 24 h. Notably, CCK8 analysis showed that NAC and SP600125 could attenuate the cell-killing effect of erianin on the growth of OS cells (Figure 5a). Flow cytometric analysis demonstrated that NAC and SP600125 attenuated the erianin-induced apoptosis (Figure 5b), and western blotting analysis showed that both of them reduced the levels of apoptosis-related proteins (Figures 5c and d). Next we investigated the role of ROS generation and JNK activation in erianin-induced autophagy. Results showed that NAC and SP600125 decreased the number of GFP-LC3 puncta (Figure 5e) and the expression of LC3-II, Beclin-1, and p62 proteins (Figures 5f and g). Taken together, ROS/JNK pathway activation by erianin participated in the induction of apoptosis and autophagy.

### Erianin inhibits growth of OS *in vivo*

**Figure 6:**
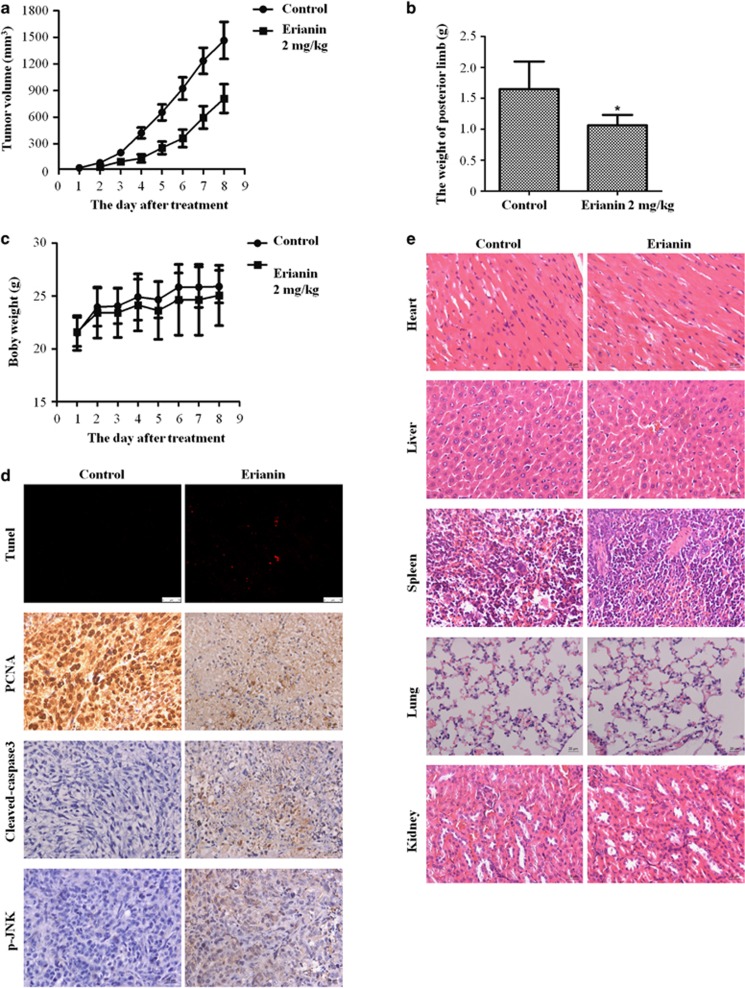
Erianin inhibits OS xenograft growth *in vivo*. 143B cells were orthotopically inoculated into the left tibia of BALB/c-nu mice. One week after tumor inoculation, mice were randomly divided into two groups for treatment. Intraperitoneal administration of vehicle or erianin (2 mg/kg) every other day for seven times. (**a**) Tumor volume was measured every week. (**b**) Erianin treatment resulted in significantly lower than control group. (**c**) Body weights were measured every week. (**d**) Detection of apoptosis in tumor tissues by TUNEL assay. The expression of PCNA (proliferating cell nuclear antigen), cleaved caspase-3, and p-JNK were examined by immunohistochemistry. (**e**) No major organ-related toxicities was observed. H&E staining was used to evaluate the histology. Data represent mean±S.D. *P<0.05, significantly different compared with control

To evaluate the antitumor effect of erianin *in vivo*, an orthotopic OS model was established by intra-tibial injection of 143B cells. The mice were injected with erianin (2 mg/kg) while control group were injected with 5% dimethyl sulfoxide (DMSO) intraperitoneally every other day for seven times in total. Erianin inhibited the growth of tumor (Figures 6a and b). However, there was no significant loss in body weight in the experimental mice (Figure 6c). As shown in Figure 6d, erianin-treated tumor tissues showed significant increase of terminal dUTP nick end labeling (TUNEL)-positive cells and the level of cleaved caspase-3 and JNK phosphorylation, whereas the level of PCNA was decreased. To investigate any potential cytotoxic effects of erianin on normal tissues, non-tumor-bearing mice were intraperitoneally treated with erianin, and hematoxylin and eosin (H&E) staining of organs collected at the end of the study also suggested no major organ-related toxicities (Figure 6e). These data showed that erianin exhibited potent antitumor activity with less toxicity *in vivo*.

## Discussion

Erianin, a natural product derived from *D. chrysotoxum*, has been associated with potent antitumor activity against human cancer cell lines tested with IC50<100 nM. Previous studies for erianin anti-tumor effects were described as followed: induced vascular shutdown, inhibited angiogenesis, disrupted endothelial tube formation, and perturbed cityscape's architectural.^9, 34^ However, for proliferating and fragile tumor cells and normal tissue endothelial cells, the mechanism of action of erianin is different. Erianin was shown to be cytotoxic toward proliferation and induction of apoptosis but not quiescent endothelial cells. Non-cytotoxic concentrations of erianin resulted in the disruption of endothelial cytoskeleton, implying that there is a cell-type specificity in erianin-induced cytoskeletal impairment.^9^ These studies suggest that erianin may have future value in the treatment of some non-cancer diseases. In the current study, we undertook a comprehensive analysis of the effect of erianin on OS using both *in vitro* and *in vivo* models. Our data showed that erianin could suppress cell proliferation, cause G2/M-phase arrest, and induce apoptosis and autophagy via the ROS/JNK signaling pathway in human OS cells.

The G2 checkpoint prevents cells from entering mitosis when DNA is damaged, providing an opportunity for repair and stopping the proliferation of damage cells.^14^ According to flow cytometric analysis, erianin increased the proportion in G2/M phase and decreased the cell proportion in G0/G1 and S phases in OS cells. Western blotting analysis showed that erianin led to an increase in the accumulation and activation of G2/M-phase-related cycle regulator Cyclin B1. However, whether erianin inhibited G2/M transition or induced M arrest has been uncertain. The complex of Cdk1/Cyclin B1 has a crucial role in promoting the G2/M-phase transition. Further analysis revealed that erianin increased the expression levels of phospho-Cdk1. In addition, we examined the effects on p21, p27, and the phospho-Cdc25c. P21 and p27 have a critical role in blocking activation of Cdk1/Cyclin B1,^35, 36, 37^ while Cdc25c is activated for dephosphorylation of cdc2 at the onset of mitosis.^38, 39^ Increased expression of p21, p27 and phospho-Cdc25c were observed after erianin treatment. These data indicated that erianin inhibited the G2/M transition, rather than causing M-phase arrest. Existing evidence shows that antitubulin agents are associated with inappropriate activation of Cdc25c and Cyclin B1, resulting in the induction of G2/M phase arrest.^40, 41^ However, the mechanism underlying this phenomenon has largely remained elusive and need to be further explored.

Apoptosis is crucially involved in the regulation of tumor formation and treatment response. Current cancer therapy, including chemotherapy, *γ*-irradiation, gene therapy, and immunotherapy, has linked to activation of apoptosis signal transduction pathway.^42, 43^ Caspases, closely associated with apoptosis, are widely expressed in an inactive proenzyme form in most cells and, once activated, can often activate other procaspases, allowing initiation of a protease cascade.^44^ Present study found that erianin induced generation of apoptotic cells through the induction of DNA fragmentation and the activation of PARP, caspase-3, -8, and -9. Immunohistochemical analysis confirmed that erianin increased cleaved caspase-3 level, and the TUNEL assay demonstrated an obvious increase in apoptosis proportion in erianin-treated tumor tissues. These data indicated that erianin induced cell apoptosis by activating both the extrinsic and intrinsic (mitochondrial) pathways.

Autophagy, which is regarded as a promising, novel strategy for enhancing antitumor efficacy of chemotherapy drugs, has been under extensive investigation. A growing body of evidence implicates a dual role of autophagy for therapeutic purposes in cancer, with response protecting cell survival or contributing to cell death.^45, 46^ We found that erianin-induced autophagy in OS cells was evidenced by an increase in the number of autophagic vesicles and enhanced conversion of LC3B-I to LC3B-II. A growing number of researches demonstrated that small compounds activate apoptosis and autophagy for antitumor chemotherapy. However, the functional relationship between apoptosis and autophagy is complex, and the two phenomena that jointly decide the fate of the cell. In our study, inhibition of autophagy by treatment with 3-MA increased erianin-induced apoptosis, indicating that autophagy induced by erianin contributed to the cell survival.

ROS is considered as an important upstream molecule in the regulation of cell death and survival in cancer. Basic levels of ROS may function as signals to promote cell proliferation and survival, whereas high levels of ROS can damage cellular components such as DNA, protein and lipids, leading to cell apoptosis and autophagy.^20^ Alterations in ROS levels have a crucial role in tumorigenesis and is recognized as the promising strategy for cancer treatment. In the present study, erianin induced a significant increase in ROS generation, while pretreatment with ROS inhibitor NAC remarkably reversed the erianin-induced inhibition of cell proliferation, apoptosis, and autophagy. Our results indicated that the induction of ROS might activate DNA damage and lead to apoptosis and autophagy in OS cells.

Growing evidence in recent years demonstrates that activation of the JNK pathway transduces oxidative stress signal to promote cell apoptosis and autophagy in response to various stress signals. JNK is primarily activated by various environmental stress, including oxidative stress, chemotherapeutic agents, and heat shock. We found that treatment with erianin induced a significant increase in JNK and c-Jun phosphorylation. Confirmed by the use of the JNK inhibitor SP600125, JNK activation is associated with the regulation of erianin-induced apoptosis and autophagy. Furthermore, ROS accumulation is involved in the activation of JNK/c-Jun pathway, while pretreatment with NAC nearly attenuated the phosphorylation of JNK and c-Jun. Together, these data showed that erianin induced apoptosis and autophagy though activation of ROS-dependent JNK/c-Jun pathway.

Our study, for the first time, identifies antitumor effects of erianin on OS *in vitro* and *in vivo* and the potential molecular mechanisms. We found that erianin significantly induced G2/M cell cycle arrest and caused cell apoptosis and autophagy regulated via ROS/JNK signaling pathway. We also demonstrated that erianin significantly decreased tumor growth in mice bearing xenografts without obvious toxicity. This study indicates that erianin is a novel antitumor drug candidate that has great potential as a promising agent for anticancer therapy in OS.

## Materials and Methods

### Cell culture

Human OS cell lines 143B and Saos2 were obtained from American Type Culture Collection (Manassas, VA, USA). MG63.2 cell was derived from the metastasis of parental MG63, as previously reported.^47, 48^ Human OS cell lines CCHO were established by Pediatric Research in MD Anderson Cancer Center. All cells were finger-printed to exclude possible contamination. All cells were cultured in high-glucose Dulbecco's Modified Eagle's Medium (Thermo, Waltham, MA, USA) supplemented with 10% fetal bovine serum (Thermo), 100 U/ml penicillin, and 100 *μ*g/ml streptomycin (Thermo) in a humidified incubator at 37 °C in 5% CO_2_.

### Reagents and antibodies

Purified erianin (>98%) was purchased from Shanghai Tauto Biotech Co., Ltd (Shanghai, China). Stock solution at 100 mM was made in DMSO (Sigma, St. Louis, MO, USA) and stored in the dark at −20 °C. NAC and SP600125 were purchased from Sigma Chemical Co. (St. Louis, MO, USA). 3-MA were purchased from Selleckchem (Houston, TX, USA). Antibodies against caspase-3, -8, and -9, PARP, Bcl-2, Bcl-xl, survivin, JNK, phospho-JNK, c-Jun, phospho-c-Jun, p38, phospho-p38, Cyclin D1, Cyclin E1, Cyclin B1, Cdk1, phospho-Cdk1, phospho-Cdc25c p27, p21, LC3B, p62, Beclin-1, and GAPDH were purchased from Cell Signaling Technology (Beverly, MA, USA).

### Cell viability assay

The effect of erianin on cell viability was determined with the CCK8 (Dojindo, Kumamoto, Japan). Cell suspensions (3 × 10^4^/ml) were seeded into 96-well plates overnight and then treated with various concentration of erianin (0, 10, 25, 50, 100 nM). Erianin was dissolved in DMSO, and the concentration of DMSO was kept at <0.05% in all wells. After 24, 48, and 72 h, 10 *μ*l CCK8 solution was added to each well, and the samples were incubated at 37 °C for 2 h before the absorbance was measured at 455 nm wave length.

### Colony-formation assay

Cells were seeded in six-well plates at a density of 500 cells per well. In the drug treatment group, the medium was changed with fresh medium containing erianin (5 and 10 nM) for about 14 days until the cells grew to visible colonies. Colonies were fixed with 4% paraformaldehyde and stained by crystal violet for 15 min at room temperature. The colonies that consisted of >50 cells were counted.

### Cell cycle analysis by flow cytometry

Cells were seeded in six-well plates with a density of 5 × 10^5^/ml and then treated with erianin at different concentrations for 24 h. After erianin treatment, the cells were harvested, washed with phosphate-buffered saline (PBS) and fixed with cold 70% ethyl alcohol at 4 °C overnight. The cells were then again washed with PBS and incubated with RNase A for 30 min followed by staining with 400 *μ*l propidium iodide for 30 min at room temperature. Cell cycle analysis was performed on the Accuri C6 (BD Biosciences, Mountain View, CA, USA).

### Morphological apoptosis

Cells were cultured at a density of 5 × 10^4^/ml per well on coverslips in six-well plates and then treated with 25 nM erianin for 24 h. After incubation, cells were washed twice with PBS, fixed with 4% paraformaldehyde for 30 min, and then stained with Hoechst 33342 solution (5 *μ*g/ml) for 10 min in the dark at 37 °C. Cells were assessed by fluorescence microscope for morphological changes of the nucleus.

### Mitochondrial membrane potential assay

The JC-1 Assay Kit (Beyotime, Beijing, China) was used to measure the alteration of mitochondrial membrane potential, according to the manufacturer's instructions. Cells were seeded in six-well plates with a density of 5 × 10^5^/ml and then treated with erianin at concentrations ranging from 0 to 50 nM for 24 h. Then 100 *μ*l of JC-1 staining solution was added into 1 ml of culture medium and incubated for 20 min at 37 °C in a CO_2_ incubator. The samples were analyzed by flow cytometry, and JC-1 aggregate was measured at the FL-2 channel and green fluorescent (both JC-1 monome) at the FL-1 channel (BD Biosciences).

### Apoptosis analysis by flow cytometry

Cells were seeded in six-well plates with a density of 5 × 10^5^/ml and then treated with erianin at concentrations ranging from 0 to 50 nM for 24 h. After erianin treatment, cells were harvested, washed twice with cold PBS and resuspended in the 1 × binding buffer. Then cells were incubated with PE-conjugated Annexin V and 7-AAD for 15 min in the dark at room temperature, and the samples were analyzed using the Accuri C6 (BD Biosciences).

### Measurement of ROS

Intracellular ROS production was detected by using the peroxide-sensitive fluorescent probe DCFH–DA. Cells were plated in six-well plates and treated with erianin at different concentrations in the absence or presence of 5 mM NAC. Cells were then incubated with DCFH–DA at a final concentration of 10 *μ*M in DMEM-h without FBS for 30 min at 37 °C and washed three times with DMEM. The level of ROS was determined by fluorescence microscopy (Leica, Wetzlar, Germany) and flow cytometer (BD Biosciences; San Jose, CA, USA).

### GFP-LC3 puncta assay

Autophagy was examined by analyzing the formation of fluorescent puncta of autophagosomes in cells transfected with GFP-LC3. Cells were cultured in six-well plates and transfected with 2 *μ*g/ml GFP-LC3 plasmid, using Lipofectamine 2000 (Invitrogen, Carlsbad, CA, USA), following the manufacturer's protocol. After 24 h posttransfection, the cells were treated with or without 25 nM erianin for different times (12 and 24 h) or with different concentrations of erianin (0, 10, and 25 nM) for 24 h. Image acquisition was performed using a fluorescence microscope.

### LysoTracker Red staining

Cells were cultured in six-well plates at a density of 5 × 10^5^/ml per well and then treated with erianin (0, 10, or 25 nM) for 24 h. The cells from different treatments were collected and then incubated with 50 nM of LysoTracker Red DND-99 (Invitrogen) in the dark for 30 min at 37 °C, and the samples were analyzed using a fluorescence microscope.

### Western blotting analysis

Cells were cultured in six-well plates at a density of 5 × 10^5^/ml per well and then treated with erianin (0, 1, 10, 25, or 50 nM) for 24 h. Cells were washed with PBS, lysed in ice-cold RIPA containing a protease and a phosphatase inhibitor cocktail for 30 min on ice. Cell lysates were centrifuged at 13 000 × *g* for 15 min at 4 °C, and the supernatant was collected. Protein concentrations were quantified using the BSA Protein Assay according to the manufacturer's instruction. Equal amounts (30 *μ*g) of total protein were separated by SDS-PAGE (8–12%) at 100 V for 1.5 h and transferred to 0.45*-μ*m PVDF membrane at 100 V for 1 h. After blocking with 5% non-fat milk in PBST buffer for 1 h at room temperature, the membranes were incubated with primary antibody at 4 °C overnight. The membranes were washed three times with PBST buffer and then incubated with peroxidase-conjugated secondary antibody for 1 h at room temperature. Specific antibody binding was detected by the Chemiluminescence Kit (Millipore, Plano, TX, USA).

### Orthotopic xenograft OS mouse model

Female BALB/c-nu mice (Shanghai Slac Laboratory Animal Co., Ltd., Shanghai, China) were purchased at 4 weeks of age and housed in a standard animal laboratory with free access to water and food. 143B cells were digested and washed by cold PBS for three times, and the final concentration was 1 × 10^7^/ml in cold PBS. A volume of 100 *μ*l cell suspension was injected into medullary cavity of tibia. When the tumors in the tibia were macroscopic, mice were randomly divided into two groups: control group and erianin group (six mice in each group). Controls group received intraperitoneal injection of 100 *μ*l 5% DMSO every other day, while erianin group was injected with 100 *μ*l erianin (2.0 mg/kg, diluted with 5% DMSO). After seven times of drug administration, the mice were killed, and the tumors were removed, weighted, and fixed for use in immunohistochemical experiments. All the animal-related procedures were approved by the Animal Care and Use Committee of The First People's Hospital of Shanghai.

### TUNEL assay

Apoptosis detection was identified using a TUNEL Assay Kit (Beyotime, Beijing, China) according to the manufacturer's instructions. In brief, paraffin-embedded slides were deparaffinized with xylene and ethanol and rehydrated cell by proteinase K. After several washes with PBS, sections were incubated with TUNEL reaction mixture prepared freshly for 1 h at 37 °C in a moist chamber. Apoptotic cells on the slides were observed under an Olympus light microscope (Olympus, Tokyo, Japan) in randomly chosen fields.

### Histopathology and immunohistochemistry

Formalin-fixed tissue samples were embedded in paraffin and 4*-μ*m sections were cut. Primary tumors, heart, liver, spleen, lung, and kidney sections were stained with H&E for routine histological examinations and morphometric analysis. For immunohistochemical staining, slides were deparaffinized in xylene and rehydrated with graded alcohol and incubated in 3% hydrogen peroxide to block the endogenous peroxidase activity. Antigen retrieval was performed by boiling the slides in 10 mM sodium citrate (pH 6.0) for 30 min. Then slides were blocked in 10% normal goat serum for 15 min, followed by incubation with PCNA, p-JNK, and cleaved caspase-3 at 4 °C overnight in a moist chamber. On the next day, slides were washed in PBS and incubated with the second antibody for 1 h at room temperature. Immunoreactivity was detected using the Vectastain Elite DAB KIT (Vector Laboratories, Burlingame, CA, USA).

### Statistical analysis

Statistical analysis was performed using the SPSS version 18.0 software (IBM Corporation, Chicago, USA). Student's *t*-test, Fisher's Exact test, and one-way ANOVA were used for calculating the significance between different groups. Statistical significance is indicated by *P*<0.05. All data were expressed as mean±S.D. of three independent experiments.

## Abbreviations

OS: osteosarcoma
ROS: reactive oxygen species
JNK: c-Jun N-terminal kinase
NAC: *N*-acetyl cysteine
3-MA: 3-methyladenine
CA-4: combretastatin A-4
PARP: poly (ADP-ribose) polymerase
PCD: programmed cell death
CCK8: Cell Counting Kit-8
TUNEL: terminal dUTP nick end labeling
H&E: hematoxylin and eosin
